# Age- and sex-specific normative references and performance zones for core muscular endurance in adolescents aged 12–16 years: a cross-sectional study

**DOI:** 10.3389/fphys.2026.1802720

**Published:** 2026-10-20

**Authors:** Guo Yanlan, Yihao Liu, Syed Ghufran Hadier, Syed Danish Hamdani, Jian Wu, Syed Muhammad Zeeshan Haider Hamdani

**Affiliations:** 1 School of Physical Education, North University of China, Taiyuan, Shanxi, China; 2 School of Sport and Health Sciences, Sichuan Technology and Business University, Chengdu, Sichuan, China; 3 Department of Sports Sciences, Bahauddin Zakariya University, Multan, Punjab, Pakistan; 4 Department of Health Sciences and Sport, University of Stirling, Stirling, United Kingdom; 5 Stirling College, Chengdu University, Chengdu, Sichuan, China

**Keywords:** back substitution procedure, core muscular endurance, fitness evaluation, LMS method, normative reference, Pakistani adolescents, performance zones, plank exercise

## Abstract

**Objective:**

This study aimed to develop age- and sex-specific normative percentile curves and percentile-based performance zones for core muscular endurance using the plank exercise test among adolescents aged 12–16 years in South Punjab, Pakistan, employing the LMS method and a back substitution procedure to assess internal consistency.

**Method:**

This cross-sectional study included 2, 970 adolescents (49.73% boys, 50.27% girls; mean age 14.00 ± 1.41 years) recruited from 60 higher secondary schools across South Punjab, Pakistan, using stratified random sampling in 2020, representing a pre-pandemic baseline. Height, weight, and BMI were recorded, and core muscular endurance was assessed using the isometric plank test. Age- and sex-specific normative references and smoothed percentile curves were established using the LMS (Lambda, Mu, Sigma) method. Internal consistency of the derived reference values was assessed using a back substitution procedure, in which the LMS model’s P50 (50th percentile) estimate for the full sample was compared against its P50 estimate for a randomly drawn 20% subsample of the same cohort, with the Mean Absolute Percentage Error (MAPE) used to quantify agreement. Sex differences were examined using the Mann-Whitney U test, with statistical significance set at p < 0.05.

**Results:**

Using the LMS method, the study established age- and sex-specific normative references and smoothed percentile curves for the plank test. Boys were significantly taller, heavier, and had higher BMI than girls (p < 0.001). Boys showed higher mean plank times (80.72 ± 73.48 s vs. 69.07 ± 58.16 s; p < 0.01), effect sizes ranged from negligible to small across all variables (rank-biserial r = 0.00–0.26), with r = 0.06 for plank performance. Median plank performance increased with age in both sexes, from 50 to 61 seconds in boys and from 44 to 66 seconds in girls between ages 12 and 16, with a steeper rise in the upper percentiles among boys. Age- and sex-specific percentile curves and performance cut-points across five zones; Very Poor, Poor, Medium, Good, and Excellent were derived. The majority of participants were classified as “Medium” (32.7%) or “Good” (24.4%), with 9.9% reaching “Excellent”. Boys’ “Excellent” threshold ranged from ≥133 seconds at age 12 to ≥227 seconds at age 16, while in girls it ranged from ≥127 to ≥126 seconds, peaking at ≥146 seconds at age 14. The back substitution procedure produced an average MAPE of 6.69% for boys and 6.04% for girls, supporting the internal consistency of the derived references within this population.

**Conclusion:**

This study provides the first age- and sex-specific normative references and percentile-based performance zones for the plank exercise test in Pakistani adolescents, with internal consistency supported by a back substitution procedure. These references offer simple, low-resource, population-based benchmarks for monitoring core muscular endurance, with practical value for physical education teachers, coaches, and health professionals working in school-based health initiatives. However, external validation using an independent sample, and replication in other regions of Pakistan, are needed before these references are applied beyond South Punjab.

## 1 Introduction

Core muscular endurance, defined as the capacity to sustain isometric muscular contraction over time, is a fundamental component of physical fitness (PF), contributing to postural stability, balance, injury prevention, and long-term musculoskeletal health (Santos et al., 2019; Marković and Milošević, 2023). Despite its importance, Pakistani adolescents face growing public health challenges that threaten PF levels, including rising rates of obesity, physical inactivity, and sedentary behaviour (Liu et al., 2023; Yang et al., 2025). Obesity rates among school-aged children in Pakistan have risen from 7.5% to 13.4% over the past decade (Liu et al., 2023). This trend bears directly on core muscular endurance, because excess adiposity adds to the mechanical load the trunk must support and is consistently associated with poorer relative muscular endurance in young people; in field testing, higher waist circumference and body-fat percentage predict shorter plank hold times and lower muscular-endurance performance in children and adolescents (Boyer et al., 2013; Garcia-Pastor et al., 2016). Alongside rising obesity, insufficient emphasis on structured physical education, increased screen time, and rapid urbanization have collectively contributed to deteriorating fitness trends among young people (Hamdani et al., 2023; Uddin et al., 2025). The absence of culturally relevant fitness assessment tools and region-specific benchmarks further limits the ability to monitor and address these concerns effectively (Hamdani et al., 2023; Hadier et al., 2024a).

Adolescence, particularly between the ages of 12 and 16 years, is a period of substantial physical change characterized by rapid musculoskeletal growth, shifts in body composition, and the refinement of motor skills (Stout, 2016). These chronological age-related changes necessitate standardized, age-appropriate fitness assessments to track physical development and guide targeted health interventions (Stout, 2016). The World Health Organization and various national health agencies emphasise the importance of regular fitness monitoring during this period to support lifelong health outcomes (Hadier et al., 2024a; Hadier et al., 2025). Establishing region-specific benchmarks is particularly critical, as genetic variation, environmental conditions, and lifestyle factors all influence physical growth and fitness outcomes differently across populations (Laurson et al., 2022). In Pakistan, the absence of population-specific fitness normative references and performance standardization makes accurate evaluation particularly difficult for educators, coaches, and health professionals alike (Hamdani et al., 2023).

Core muscular endurance plays a central role in adolescent health and physical functioning. Adequate core endurance supports the trunk and spine, improving balance and movement efficiency (Laurson et al., 2017), while also providing a protective effect against lower back pain, an increasingly prevalent concern among adolescent populations (Fawaz et al., 2024). During the ages of 12 to 16 years, core endurance is particularly important for injury prevention and postural stability, as the body undergoes rapid structural changes that place greater demands on the musculoskeletal system (Akuthota et al., 2008; Granacher et al., 2013). Furthermore, because core endurance capacities differ between sexes across this age range, age- and sex-specific benchmarks are essential for accurate fitness evaluation and the design of targeted interventions (Hottenrott et al., 2021; Khurram, 2026).

Traditional laboratory-based methods for assessing core muscular endurance, such as isokinetic dynamometry and electromyographic analysis, are frequently impractical in school and community settings due to their high cost, technical complexity, and time demands (Janz et al., 2022). The isometric plank test offers a simple, cost-effective, and field-applicable alternative, requiring only a stopwatch and a soft mat, making it well suited for large-scale population assessments (Henriques-Neto et al., 2020; Hadier et al., 2024b). Its utility in youth populations is well established internationally: the U.S. National Youth Fitness Survey (NYFS) recognizes the plank test as a core endurance benchmark for children and adolescents aged 3–15 years (CDC, 2012), and health-referenced performance references for the plank have been developed and validated in school-based settings (Institute of Medicine, 2012; Chang et al., 2020). Normative reference data for plank performance in adolescents have also been reported in North American and European populations (Tomkinson et al., 2018; Lopes et al., 2021; Day et al., 2022; Laurson et al., 2022). Conceptually, the present study is grounded in the health-related fitness surveillance framework, in which population-specific, age- and sex-referenced norms provide the interpretive basis for identifying adolescents with low fitness and for guiding targeted intervention, the same logic that underpins established youth-fitness systems such as FitnessGram and the Canadian Assessment of Physical Literacy (Day et al., 2022; Hadier et al., 2024a). Within this framework, however, no age- and sex-specific normative references for the plank test currently exist for Pakistani adolescents, representing a significant gap in regional fitness data.

In South Punjab specifically, socioeconomic diversity and limited healthcare infrastructure have contributed to a pronounced scarcity of localized fitness data, further undermining the effectiveness of public health and physical education initiatives (Mushtaq et al., 2012; Hadier et al., 2025). The LMS (Lambda, Mu, Sigma) method is particularly well suited for developing such normative references. Unlike empirical percentile ranking, which is sensitive to sampling fluctuation and can produce irregular curves across adjacent age groups, the LMS method models skewness (L), the median (M), and the coefficient of variation (S) as smooth functions of age, yielding continuous, age- and sex-specific percentile curves that remain statistically robust even where subgroup sizes are modest (Long et al., 2024; Yang et al., 2025; Khurram, 2026). For this reason, it has become an established approach for constructing growth and fitness reference curves in paediatric and adolescent populations. Back substitution procedure complements this approach by providing an internally consistent check on the LMS-derived normative references, offering an evidence-based basis for their practical application prior to external validation (Khurram, 2026).

Against this background, a clear need exists for population-specific reference values that allow plank performance to be interpreted meaningfully within the Pakistani adolescent population, rather than against norms derived from structurally different populations. The present study therefore aimed to develop age- and sex-specific normative percentile curves for plank performance among adolescents aged 12–16 years in South Punjab, Pakistan, using the LMS method, and to examine their internal consistency through a back substitution procedure. From these curves, percentile-based performance zones were derived to translate continuous hold times into interpretable fitness categories. Because the plank test requires minimal equipment and little administration time, the resulting references offer physical education teachers, coaches, and health professionals a practical basis for identifying adolescents with low core endurance, tracking change across a school year, and prioritizing those most likely to benefit from intervention. In doing so, the study addresses a capability gap created by the current absence of population-specific norms and contributes to the wider evidence base for adolescent fitness surveillance in Pakistan and comparable low-resource settings.

## 2 Methods

This cross-sectional study was conducted in high schools across South Punjab, Pakistan, and data were collected in 2020. Reporting follows the Strengthening the Reporting of Observational Studies in Epidemiology (STROBE) guidance for cross-sectional studies, and the completed checklist is provided as a supplementary file. The South Punjab Province was divided into three geographical strata based on administrative divisions: Multan, Bahawalpur, and D.G. Khan. Using a stratified random sampling approach with equal allocation, 20 schools were randomly selected from each division, representing approximately 16.67% of the total 360 schools (Hadier et al., 2024a; Liu et al., 2026). Within each selected school, 50 students were randomly chosen to ensure equal opportunity and maintain representativeness. This approach was chosen to include a diverse range of socioeconomic and cultural backgrounds, thereby enhancing the generalizability of the findings.

### 2.1 Sampling

The sample size for this study was determined using formula for sample size calculation as follows $n=\frac{{\left(Z\right)}^{2}PQ}{e^{2}}\times D$ (Cochran, 1977). In this equation, P was assumed as 0.4 based on regional school enrollment patterns in the absence of prior normative data, with Q = 0.6. The Z value was set at 1.96 for a 95% confidence level, e = 0.05 for precision, and the design effect (D) was assumed to be 1, as stratification was used for representation while selection within strata remained random. Although this calculation yielded a minimum required sample size of 369, the study deliberately targeted a substantially larger sample (approximately 3, 000 adolescents aged 12–16 years) to improve precision, subgroup representation by age and sex, and the robustness of the normative reference values. This approach is consistent with recommendations for large-scale normative studies. This larger sample size was chosen to align with the objectives of achieving broader generalizability, as noted in previous research (Malina, 2001; Hadier et al., 2024c).

Using an equal allocation method, the sample was balanced between boys and girls (approximately 50% each) to ensure comparability across sex groups and ensure a balanced representation (Hamdani et al., 2023; Liu et al., 2023). After accounting for non-responsive or incomplete responses, the final sample included 2970 participants aged 12-16. This South Punjab adolescent cohort has previously supported normative reference publications on anthropometric growth (Long et al., 2024) and handgrip strength (Yang et al., 2025). The present study draws on the same cohort but reports an entirely separate outcome, core muscular endurance assessed by the plank test, which was not analysed, modelled, or reported in either prior publication; it therefore contributes the first age- and sex-specific plank reference values and performance zones for this population, extending the cohort’s normative work from body size and grip strength to trunk muscular endurance. Core muscular endurance is a distinct component of health-related fitness, with associations to spinal stability and postural control that differ from those of morphological growth or maximal grip strength, and the present study yields a separate practical output in the form of age- and sex-specific performance zones for screening. The three publications therefore address different fitness constructs within a shared cohort and are individually interpretable. Targeting adolescents aged 12–16 allowed the study to capture characteristics pertinent across early to mid adolescence, thereby providing a comprehensive view of adolescent developmental difference across this crucial age span. This strategy ensures a balanced and representative insight into adolescent growth and behaviors. **Figure 1** shows the study sampling and complete procedure of the study.

**Figure 1:**
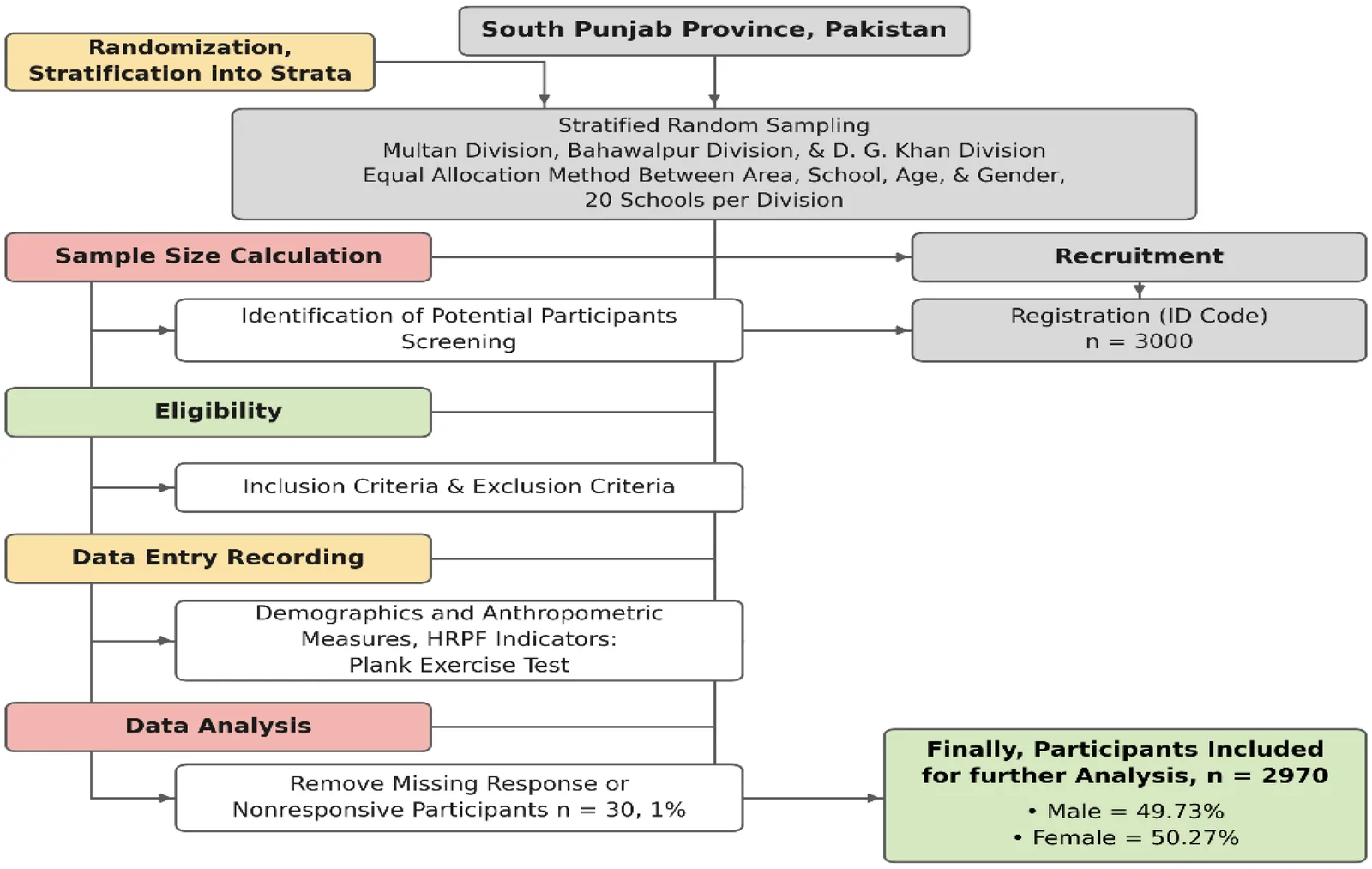
Flow chart of study sampling. HRPF, health-related physical fitness; ID, identification; n, number of participants

### 2.2 Participation criteria

Participants included in this study were adolescents aged between 12 and 16 years. Eligibility was determined through a structured screening process using available school health records, participant health information, and field-based screening at the time of recruitment. All participants were required to be physically capable of performing the fitness test without assistance. Exclusion criteria were determined using student self-report (physical disabilities, severe chronic conditions and injuries) and teacher confirmation prior to testing. Students with any physical disabilities, severe chronic conditions that could impair test performance, or with recent significant injuries were excluded from the study.

### 2.3 Ethical approval

This study received ethical approval from the Research Ethics Committee of Bahauddin Zakariya University, Multan, Pakistan (approval number 357/UREC/20). Before data collection, written and verbal consent was obtained from participating students, parents/guardians, and school management. The study was conducted in full accordance with the Declaration of Helsinki, ensuring adherence to national and international ethical references for human research.

### 2.4 Measures and procedure

The anthropometric measurements collected were height, weight, and BMI, selected for their simplicity and effectiveness in assessing growth. Additionally, core muscular endurance was evaluated using the Plank exercise test to account for endurance performance. Biological maturation status (e.g., Tanner stage or peak height velocity) was not assessed; in keeping with established field-based fitness reference systems such as Eurofit and the MoMo study, norms were constructed using chronological age and sex, the variables most readily available to teachers and practitioners in school settings (Tomkinson et al., 2018; Niessner et al., 2020).

#### 2.4.1 Demographics and anthropometric measures

Demographic data, including age, sex, and living area, were collected through student self-reports or school records.

#### 2.4.2 Height

Height was measured to the nearest 0.1 cm using a height-weight scale (DT-150 Height and Weight Scales, Shanghai, China) with the metrical rod of this scale used to measure height. Participants stood barefoot against a wall on a flat surface, maintaining a straight posture with arms relaxed, heels together, and toes slightly apart at a 60-degree angle.

#### 2.4.3 Weight

Weight was measured in kilograms using the same height-weight scale. Accuracy was verified with 100g and standard weights, ensuring errors did not exceed 0.1 kg. Participants stood barefoot on the scale, and their weight was recorded in one decimal place.

#### 2.4.4 Body mass index

BMI was calculated by dividing weight in kilograms by the square of height in meters. BMI is an internationally recognized standard for measuring obesity and assessing health, providing a consistent means to analyze the impact of weight across individuals of varying heights (CDC, 2020). The formula used is: $BMI=\frac{\text{Weight }(\text{kg})}{\left[\text{Height }{\left(m\right)}^{2}\right]}$.

#### 2.4.5 Core muscular endurance

The plank exercise test was used to assess core muscular endurance, a widely recognized and validated measure in adolescent populations (Boyer et al., 2013). Prior to formal testing, a standardized familiarization trial was conducted to ensure that all participants understood the correct plank technique and the testing procedure. Each participant received verbal instructions and a visual demonstration and was permitted to make a brief practice attempt before data collection commenced. This approach was implemented to improve measurement consistency and reduce performance variability attributable to task unfamiliarity.

Before the test itself, participants completed a standardized dynamic warm-up of approximately two and a half minutes, designed to activate the core, shoulder, and leg muscle groups involved in maintaining correct plank posture. The warm-up comprised 30 seconds each of jogging in place, leg swings, arm circles, walking lunges, and hip rotations. Following the warm-up, participants assumed a forearm plank position on a flat mat. The forearms were placed on the ground with the elbows bent at 90 degrees and positioned directly beneath the shoulders, hands unclasped. Feet were set hip-width apart with ankles at 90 degrees. Participants were instructed to maintain a straight body alignment from head to heels, a neutral pelvis, and an engaged core throughout the duration of the test.

Standardized monitoring and correction procedures were applied consistently across all testing sessions. The evaluator gave a verbal warning at the first sign of incorrect form, such as hips dropping or lifting, back bending or shifting, or poor arm and foot position, and allowed the participant to adjust without stopping the timer. A second mistake led to another opportunity to correct the position. The test ended after a third mistake or when the participant stopped due to fatigue. Participants were asked to sustain the plank position for as long as possible, with the test continuing until volitional fatigue or until termination criteria were met. No fixed upper time limit was imposed. Although some prior protocols have applied a maximum hold time (Boyer et al., 2013), the present study deliberately recorded performance to the point of fatigue in order to capture the full range of core endurance, including high-performing adolescents whose capacity would otherwise be truncated. Total hold time was recorded in seconds using a stopwatch. This open-ended protocol allowed the upper percentiles of the distribution to be characterized directly from observed data rather than estimated against an artificial ceiling.

Although direct test-retest or inter-rater reliability was not assessed within the present study, the plank test has demonstrated moderate to good reliability in comparable youth populations under standardized conditions [ICC ≈ 0.60–0.85 (Zouita Ben Moussa et al., 2020; Hadier et al., 2025)]; these numbers are cited as supportive evidence rather than as confirmatory data for the present sample. The combination of a trained research team, a structured warm-up, a pre-test familiarization trial, and consistent termination criteria supports the measurement consistency of the data obtained.

### 2.5 Study procedure

Prior to data collection, all necessary approvals and consents were obtained from school principals and parents, adhering to ethical references for research involving young population. Strict privacy measures were implemented, including prohibiting photography and establishing sex-specific guidelines for research assistants. Data collection was conducted during the school Physical Education lessons in collaboration with school administrators to ensure alignment with the school’s academic schedule, thereby minimizing disruptions to regular educational activities. Each participant was tested individually to maintain the accuracy and consistency of measurements. A team of 12 research assistants from the Department of Sports Sciences at Bahauddin Zakariya University was assembled. A rigorous training workshop was conducted for all research assistants before data collection to address inconsistencies between evaluators. The workshop included standardized instruction on correct plank positioning, repeated practice trials to establish consensus on verbal correction sequences, and supervised pilot testing to confirm consistent application of the three-mistake termination rule across all assessors. This training was designed to ensure standardization of measurement techniques in accordance with the protocols established in previous research in this population (Hadier et al., 2024c; Peng et al., 2026).

Data were collected in various schools across the South Punjab province, Pakistan. Each school visit was carefully planned to align with available class schedules, and testing requirements were communicated to school officials two days in advance. Two team members visited each school, collaborating with the school’s physical education teacher to facilitate data collection. On the day of data collection, students aged 12–16 were randomly selected for participation. Initial measurements included height and weight, calculation of BMI, and collection of demographic details such as school, class, sex, and date of birth from school records. Subsequently, plank exercise test performance was evaluated by the two evaluators with the assistance of the school PE teacher. All assessments were completed within a single working day.

### 2.6 Establishing normative references and performance zones

This study develops age- and sex-specific normative references and percentile-based performance zones for the plank test, a widely used measure of core muscular endurance, in adolescents aged 12–16 years. Using the LMS method, normative reference values and percentile curves were created to provide an evaluation framework that accounts for age and sex differences. The internal consistency of these references and percentile curves was then assessed using the back substitution procedure.

#### 2.6.1 LMS method and its application

The Lambda-Mu-Sigma (LMS) method, originally proposed by Cole and Green (Cole, 1990), was used to construct age- and sex-specific normative reference curves for plank performance. The method models three parameters Lambda (L, skewness), Mu (M, median), and Sigma (S, coefficient of variation) and is widely used in growth and fitness research for its ability to accommodate non-normal, age-varying data (Niessner et al., 2020; Zouita Ben Moussa et al., 2020; Bchini et al., 2025). Curves were fitted separately for each sex using the Box-Cox Cole-Green (BCCG) distribution within the GAMLSS framework in R. Because chronological age was recorded in completed years, the reference curves are supported by only five discrete age points across the 12–16-year range; each of the three parameters was therefore modelled as a quadratic function of age, providing an adequate, parsimonious fit given this age resolution. Model adequacy was confirmed using worm plots, Q–Q plots, and inspection of the normalised quantile residuals, which conformed closely to the standard normal distribution for both sexes (mean ≈ 0, variance ≈ 1, skewness ≈ 0; **Supplementary Figure S1**).

Percentile values were derived from the fitted L, M and S parameters using the standard LMS quantile equations:

C = M × (1 + L × S × Z)^(1/L), when L ≠ 0

C = M × exp(S × Z), when L = 0

where C is the percentile value in seconds and Z is the standard normal deviate corresponding to the desired percentile. Because the BCCG distribution transforms the outcome to normality through the Box-Cox power L, the normal deviate is the appropriate quantile function, and the three tabulated parameters are together sufficient to reproduce any reported percentile. This approach allows the generation of continuous, age-specific normative reference values that reflect the distributional characteristics of the study population.

#### 2.6.2 Interpretation of performance zones

A five-zone classification framework, with boundaries at the 10th, 35th, 65th, and 90th percentiles, was adopted from the Chinese National Physical Fitness Evaluation Standard (Jiang, 2004), a large-scale national system widely used for youth fitness grading. This framework was preferred over binary healthy-zone classifications because it provides finer discrimination across the performance distribution, identifying not only adolescents with low core endurance but also those at intermediate and high levels, information directly useful for tracking change and tailoring intervention. Performance zones were defined as Very Poor (≤ P10), Poor (P10–P35), Medium (P35–P65), Good (P65–P90), and Excellent (≥ P90), with higher zones reflecting greater core muscular endurance. Cut points were derived separately for each age and sex group to ensure comparability across developmental stages.

#### 2.6.3 Internal consistency check using the back substitution procedure

The internal consistency of the LMS-derived normative references was evaluated using the Back substitution procedure described by Khurram (2026). The procedure assesses the stability of the generated references rather than measurement reliability. The LMS model was fitted once to the complete sample (n = 1, 477 boys; n = 1, 493 girls) to generate the smoothed references and percentile curves. A random subsample of approximately 20% of participants from each sex (n = 295 boys; n = 299 girls) was then drawn from the same dataset, and the LMS-derived median (P50) was obtained separately for each age and sex group from the complete dataset and from the subsample. The full-sample estimates served as the reference values and the subsample estimates as the comparison values. Agreement between the two sets of LMS-derived P50 estimates was quantified using the Mean Absolute Percentage Error (MAPE), as defined in the source methodology (Khurram, 2026): MAPE = (1/n) × Σ |(Reference estimate − Comparison estimate)/Reference estimate|×100.

where n is the number of age strata. A MAPE below 10% indicates that estimates are consistent and generalizable for the reference population (Khurram, 2026). The same modelling and back substitution approach has been applied and peer-reviewed in our previous normative work in this population (Hamdani et al., 2023). The Back substitution procedure is therefore can be interpreted as an internal consistency check on the central tendency of the derived references, comparing the fitted model’s own output across the full sample and a randomly drawn subset of it, rather than as an external criterion validation against an independently collected sample, a different population, or a health outcome.

### 2.7 Statistical analysis

Descriptive statistics are presented as means and standard deviations. The Kolmogorov–Smirnov test indicated that plank performance was not normally distributed, so sex differences were examined using the Mann–Whitney U test, with the rank-biserial correlation (|r|) reported as the effect size, Benjamini-Hochberg false discovery rate correction was applied separately within **Tables 1**, **2**, and q-values are reported alongside p-values. Statistical significance was set at p < 0.05. Outlier detection was conducted within age- and sex-specific subgroups using a conservative threshold of ±5 standard deviations, chosen to retain plausible extreme values in a large normative dataset, since stricter criteria can distort percentile estimation at the tails. No values exceeded this threshold.

**Table 1:** Sex-specific descriptive analysis of anthropometric characteristics.

Component	Total (n=2970)	Boys (n=1477)	Girls (n=1493)	p-value (|r|)	q-value
Age (years)	14.00 ± 1.41	14.00 ± 1.41	14.00 ± 1.42	0.989 (r=0.00)	0.989
Height (cm)	159.53 ± 10.51	160.50 ± 11.50	158.57 ± 9.34	<0.001 (r=0.158)	<0.001
Weight (kg)	43.00 ± 9.10	45.02 ± 9.78	41.00 ± 7.89	<0.001 (r=0.264)	<0.001
BMI (kg/m2)	16.80 ± 2.67	17.30 ± 2.41	16.29 ± 2.82	<0.001 (r=0.244)	<0.001
Plank (sec)	74.87 ± 66.47	80.72 ± 73.48	69.07 ± 58.16	<0.01 (r=0.057)	0.009

Data are mean ± SD. Effect size is the rank-biserial correlation (|r|) for the Mann-Whitney U test. The effect-size range across variables is 0.00–0.26. q-values are Benjamini-Hochberg adjusted across the five comparisons in this table.

**Table 2:** Age and sex specific plank exercise performance.

Age	Boys mean ± SD	Girls mean ± SD	p-value	q-value	|r|
12	65.12 ± 49.24	64.88 ± 65.08	0.045	0.075	0.10
13	75.72 ± 66.04	67.00 ± 66.12	0.004	0.019	0.14
14	74.36 ± 64.96	71.35 ± 64.97	0.577	0.628	0.03
15	94.02 ± 78.73	70.69 ± 50.72	0.009	0.023	0.12
16	94.50 ± 96.14	71.48 ± 39.05	0.628	0.628	0.02

Values are mean ± SD in seconds. Effect size is the rank-biserial correlation (|r|) for the Mann-Whitney U test. q-values are Benjamini-Hochberg adjusted across the five age strata.

Age- and sex-specific smoothed percentile curves for plank performance were derived using the LMS method (Cole, 1990), fitted separately for each sex with the Box-Cox Cole-Green (BCCG) distribution within the GAMLSS framework in R (version 4.3.4, GAMLSS 5.5-4). Because age was recorded in completed years, the curves are supported by five discrete age points, and the three parameters (L, M and S) were each modelled as a quadratic function of age. Percentile values were obtained from the fitted parameters using the standard LMS quantile equations with the standard normal deviate. Model adequacy was assessed using worm plots, Q–Q plots and normalised quantile residuals, which conformed closely to the standard normal distribution for both sexes (**Supplementary Figure S1**).

Sampling uncertainty in the derived percentiles was quantified by non-parametric bootstrap. Within each sex, the full sample was resampled with replacement 1, 000 times, the model refitted to each replicate, and all percentiles recomputed; 95% confidence intervals were taken as the 2.5th and 97.5th percentiles of the resulting distributions. All replicates converged, and interval estimates are reported in **Supplementary Table S2**. The internal consistency of the LMS-derived references was examined using the back substitution procedure. All descriptive and non-parametric analyses were performed in SPSS version 21.0.

## 3 Results

The study sample comprised 2, 970 adolescents aged 12–16 years, of whom 1, 477 (49.73%) were boys and 1, 493 (50.27%) were girls. Participants were distributed approximately equally across all five age groups, as presented in **Table 3**.

**Table 3:** Respondent distribution analysis.

Age (years)	Boys N (%)	Girls N (%)	Total N (%)
12	295 (9.93)	301 (10.13)	596 (20.07)
13	295 (9.93)	298 (10.03)	593 (19.97)
14	298 (10.03)	296 (9.97)	594 (20.00)
15	298 (10.03)	300 (10.10)	598 (20.13)
16	291 (9.80)	298 (10.03)	589 (19.83)
Total	1477 (49.73)	1493 (50.27)	2970 (100)

Percentages are expressed as a proportion of the total sample (N = 2970).

**Table 1** presents the sex-specific descriptive characteristics of the sample. No significant difference was observed in mean age between boys and girls (14.00 ± 1.41 vs. 14.00 ± 1.42 years; p = 0.989, q = 0.989). Boys were significantly taller (160.50 ± 11.50 cm vs. 158.57 ± 9.34 cm; p < 0.001), heavier (45.02 ± 9.78 kg vs. 41.00 ± 7.89 kg; p < 0.001), and had a higher mean BMI (17.30 ± 2.41 vs. 16.29 ± 2.82 kg/m²; p < 0.001). Boys also recorded significantly higher mean plank times than girls (80.72 ± 73.48 vs. 69.07 ± 58.16 seconds; p < 0.01, q = 0.009). Effect sizes ranged from negligible to small (r = 0.00 to 0.26), indicating that the practical magnitude of the observed sex differences was modest. Among variables showing significant sex differences, the smallest effect was observed for plank performance (r = 0.06).

**Table 2** presents age- and sex-specific plank performance among adolescents aged 12–16 years. Boys recorded higher mean plank times than girls at each age; however, statistically significant sex differences after Benjamini-Hochberg adjustment were observed only at ages 13 and 15, with no consistent age pattern and negligible to small effect sizes throughout. At age 12, boys achieved a mean plank time of 65.12 ± 49.24 s compared with 64.88 ± 65.08 s for girls (p = 0.045, q = 0.075, |r| = 0.10, not significant after adjustment). At age 13, the corresponding values were 75.72 ± 66.04 s for boys and 67.00 ± 66.12 s for girls (p = 0.004, q = 0.019, |r| = 0.14). At age 15, boys also demonstrated higher performance than girls (94.02 ± 78.73 s vs. 70.69 ± 50.72 s; p = 0.009, q = 0.023, |r| = 0.12). No significant sex differences were found at age 14 (p = 0.577, q = 0.628, |r| = 0.03) or age 16 (p = 0.628, q = 0.628, |r| = 0.02). Overall, the effect sizes ranged from negligible to small, indicating that the magnitude of the sex differences in plank performance was limited across all age groups. Notably, the girls’ variability decreased sharply with age (SD falling from 65.08 s at age 12 to 39.05 s at age 16) while their mean changed little. As the distribution became less right-skewed, the fitted median rose toward the mean, so the girls’ rising median reflects this change in distributional shape rather than a uniform developmental gain.

**Table 4** presents the age- and sex-specific results of the back-substitution procedure for the plank exercise test. Among boys, MAPE values ranged from 1.12% to 10.21%, with an average of 6.69%. The closest agreement between the reference and comparison estimates was observed at age 12 (50.44 vs. 51.00 s; MAPE = 1.12%). The discrepancy increased progressively with age, reaching its highest value at age 16 (61.33 vs. 55.07 s; MAPE = 10.21%). Among girls, MAPE values ranged from 4.41% to 7.19%, with an average of 6.04%. The lowest MAPE was observed at age 12 (43.84 vs. 41.91 s; MAPE = 4.41%), whereas the highest occurred at age 14 (51.05 vs. 47.38 s; MAPE = 7.19%). Overall, the average MAPE was below 10% for both sexes, indicating generally close agreement between the reference and comparison estimates. All age-specific values were below 10% except among 16-year-old boys, for whom the MAPE was 10.21%.

**Table 4:** Age- and sex-specific results of the Back substitution procedure for the plank exercise test (seconds).

Age	Reference estimate (full sample)	Comparison estimate (20% subsample; boys n = 295, girls n = 299)	MAPE (%)
Boys			
12	50.44	51.00	1.12
13	54.64	52.21	4.46
14	57.86	53.29	7.91
15	60.09	54.24	9.73
16	61.33	55.07	10.21
Average	—	—	6.69
Girls			
12	43.84	41.91	4.41
13	46.48	43.45	6.52
14	51.05	47.38	7.19
15	57.54	53.69	6.68
16	65.96	62.39	5.41
Average	—	—	6.04

MAPE, Mean Absolute Percentage Error = (1/n) × Σ |(Reference estimate − Comparison estimate)/Reference estimate| × 100, where n is the number of age strata. Lower MAPE values indicate closer agreement between the reference and comparison estimates. The average MAPE was below 10% for both sexes.

The two sets of estimates also differed in the shape of the age trend: reference values increased by 4.20, 3.22, 2.23 and 1.24 s per year across the boys’ age range, whereas comparison values increased by 1.21, 1.08, 0.95 and 0.83 s. The comparison estimate was lower than the reference estimate in nine of the ten age-by-sex cells. These results therefore indicate acceptable agreement in the level of the estimates at each age, with all age-specific MAPE values at or below 10.21%, but not in the shape of the age trend, which the subsample did not reproduce.

Age- and sex-specific smoothed percentile curves were constructed using the LMS method; **Table 5** reports the L, M and S parameters with the 3rd, 10th, 35th, 50th, 65th and 90th percentiles, and **Figure 2** displays the fitted curves. The median (P50) increased with age in both sexes, from 50 to 61 seconds in boys and from 44 to 66 seconds in girls, with a steeper rise in girls across the second half of the age range. No sex difference was detected at age 14 or age 16 (q = 0.628 at both ages), and the fitted medians converge at the oldest age.

**Table 5:** Percentiles for plank exercise (seconds) by age and sex.

Age	L	M	S	P3	P10	P35	P50	P65	P90
Boys									
12	0.094	50.44	0.787	10	17	37	50	68	132
13	0.035	54.64	0.787	12	20	40	55	74	147
14	0.015	57.86	0.829	12	20	42	58	80	166
15	0.035	60.09	0.922	10	18	42	60	86	191
16^a^	0.095	61.33	1.082	6	14	40	61	92	226
Girls									
12	-0.157	43.84	0.757	12	18	33	44	59	126
13	-0.131	46.48	0.822	11	17	34	46	64	144
14	-0.007	51.05	0.812	11	18	37	51	70	145
15	0.215	57.54	0.730	11	20	43	58	76	135
16^a^	0.535	65.96	0.597	12	25	52	66	82	125

L, lambda (skewness); M, mu (median); S, sigma (coefficient of variation). Percentile values are reported in whole seconds; ᵃ Bootstrap intervals are wider at age 16 than at ages 12 to 15 (**Supplementary Table S2**); these cut-points should be applied with additional caution.

**Figure 2:**
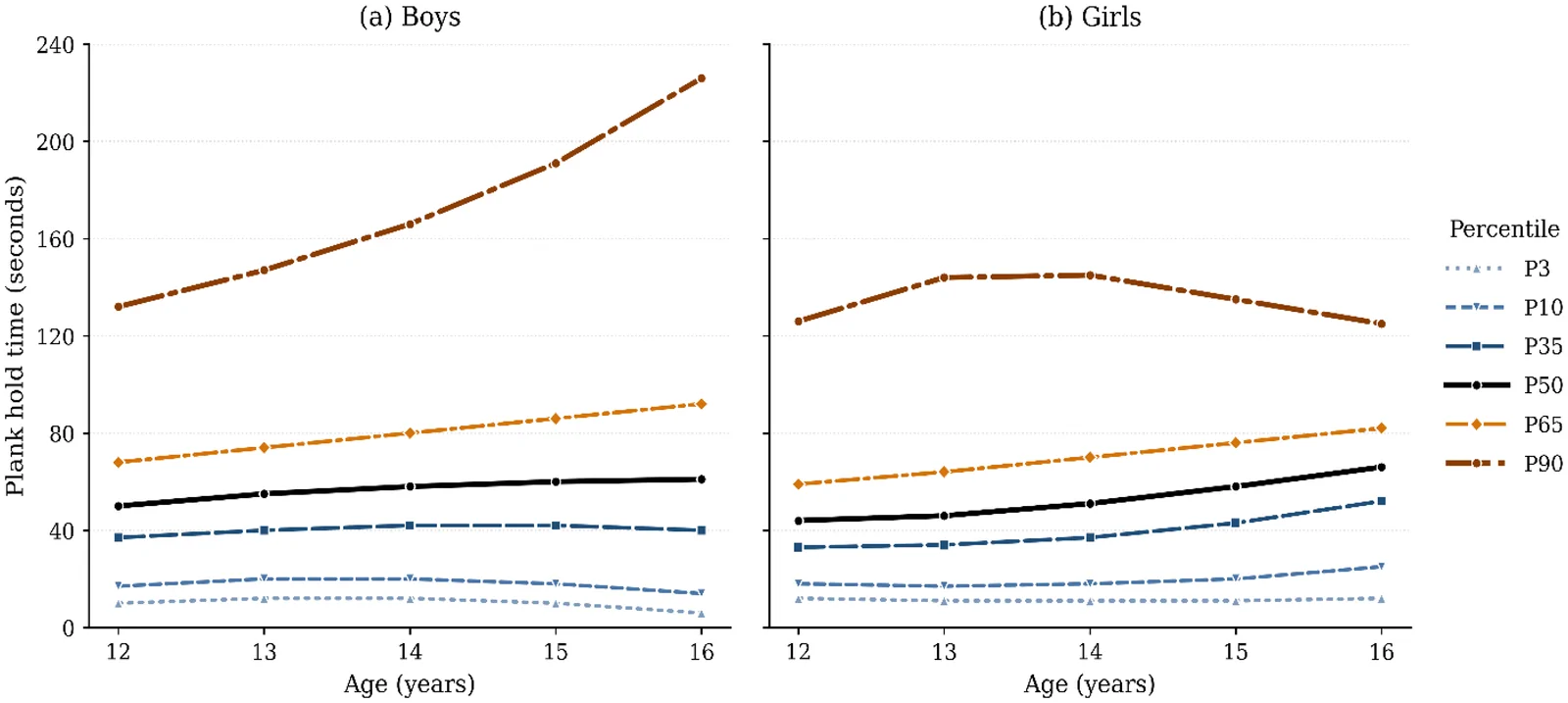
LMS-smoothed percentile curves for plank performance (seconds) in adolescents aged 12–16 years, shown separately for **(A)** boys and **(B)** girls. Curves show the 3rd, 10th, 35th, 50th, 65th, and 90th percentiles, labelled at the right margin; the horizontal axis is chronological age (completed years) and the vertical axis is plank hold time (seconds). Both panels share a common vertical scale (0–240 s) to permit direct visual comparison between sexes. A participant’s position between curves indicates their standing in the age- and sex-specific reference distribution and maps onto the performance zones in **Table 6** (Very Poor ≤ P10, Poor P10–P35, Medium P35–P65, Good P65–P90, Excellent ≥ P90).

Bootstrap confidence intervals for all tabulated percentiles are given in **Supplementary Table S2**. For most estimates the interval spans 11–20% of the fitted value. Precision was lowest at age 16 in both sexes, where the quadratic age term is least constrained. The widest interval among boys was the 90th percentile at age 16 (226 seconds, 95% CI 200–252). Among girls, imprecision at age 16 was concentrated at the lower percentiles (10th, 18–28 seconds; 3rd, 4–15 seconds), and the 3rd percentile, with a relative interval width of 92%, should be regarded as indicative only rather than as a basis for individual classification. The age-16 cut-points therefore warrant greater caution than those at ages 12 to 15.

Model-based and empirical percentiles are compared in **Supplementary Table S3**; agreement was close at the lower and central percentiles and looser at the 90th. Because hold time was recorded in whole seconds and values cluster at the floor of the scale, observed zone occupancy departs modestly from the nominal proportions at the boundary ages.

At the upper end, the 90th percentile rose distinctly in boys, from 132 seconds at age 12 to 226 at age 16, reflecting an increasingly right-skewed distribution as a subset of boys achieved very long hold times. In girls it rose from 126 seconds at age 12 to 145 at age 14, then fell to 125 at age 16. This turnover should be read cautiously: the intervals at ages 14 and 16 are only marginally separated (134–158 and 118–133 seconds), and the curves rest on five whole-year age points and are least constrained at the boundaries, so it is better understood as a boundary effect than as an established decline among older girls. The widening gap between the median and the upper percentiles in both sexes reflects the pronounced right skew within each age stratum. These are estimates from fitted reference curves, not observed longitudinal change, and describe the modelled distribution rather than individual improvement or decline over time.

**Figure 2** presents LMS-smoothed percentile curves for plank performance among boys and girls aged 12–16 years. Among boys, the median (P50) increased gradually from approximately 50 seconds at age 12 to 61 seconds at age 16. The upper percentiles also increased, particularly P90, which rose from approximately 132 to 226 seconds. In contrast, the lower percentiles changed only modestly and declined slightly at age 16. Among girls, the median and middle percentiles increased steadily with age, with P50 rising from approximately 44 seconds at age 12 to 66 seconds at age 16. The P90 curve followed a curvilinear pattern, increasing to a peak at approximately 13–14 years before declining to around 125 seconds at age 16. The lower percentiles remained comparatively stable, although P10 increased slightly at older ages. Overall, the curves indicate sex-specific differences across the plank-performance distribution. Boys showed a particularly pronounced increase at the upper end of the distribution, whereas girls demonstrated more consistent improvement across the middle percentiles. As these are cross-sectional LMS-smoothed reference curves, they should not be interpreted as longitudinal changes within individuals.

**Table 6** presents age- and sex-specific cut-points for classifying plank performance as Very Poor, Poor, Medium, Good, or Excellent. Among boys, the boundaries for the middle and upper categories generally increased with age. The Excellent threshold rose progressively from ≥133 seconds at age 12 to ≥227 seconds at age 16. In contrast, the Very Poor cut-point increased slightly from ages 12 to 14 before declining to ≤14 seconds at age 16. Among girls, the lower and middle category thresholds generally increased with age. For example, the upper boundary of the Medium category rose from 59 seconds at age 12 to 82 seconds at age 16. However, the Excellent threshold followed a curvilinear pattern, increasing from ≥127 seconds at age 12 to ≥146 seconds at age 14, before declining to ≥126 seconds at age 16. Overall, the cut-points demonstrate distinct age- and sex-specific patterns across the plank-performance distribution.

**Table 6:** Age and sex-specific performance zone cut-points for the plank exercise test (seconds).

Age	Very poor (≤P10)	Poor (P10–P35)	Medium (P35–P65)	Good (P65–P90)	Excellent (≥P90)
Boys					
12	≤17	18–37	38–68	69–132	≥133
13	≤20	21–40	41–74	75–147	≥148
14	≤20	21–42	43–80	81–166	≥167
15	≤18	19–42	43–86	87–191	≥192
16	≤14	15–40	41–92	93–226	≥227
Girls					
12	≤18	19–33	34–59	60–126	≥127
13	≤17	18–34	35–64	65–144	≥145
14	≤18	19–37	38–70	71–145	≥146
15	≤20	21–43	44–76	77–135	≥136
16	≤25	26–52	53–82	83–125	≥126

Zones are bounded by the 10th, 35th, 65th and 90th percentiles.

**Table 7** presents the distribution of participants across the five plank performance zones. Overall, the largest proportion was classified as Medium (972, 32.7%), followed by Good (724, 24.4%), Poor (612, 20.6%), Very Poor (369, 12.4%), and Excellent (293, 9.9%). Together, the Medium and Good categories accounted for 57.1% of the sample. Among boys, 30.8% were classified as Medium and 25.7% as Good, while 13.9% were Very Poor, 19.1% Poor, and 10.4% Excellent. Among girls, 34.6% were classified as Medium and 23.0% as Good, with 10.9% in the Very Poor, 22.1% in the Poor, and 9.3% in the Excellent categories. Descriptively, girls were more frequently classified as Poor or Medium, whereas boys had higher proportions in the Very Poor, Good, and Excellent categories.

**Table 7:** Distribution of participants across performance zones by sex.

Zone	Total N (%)	Boys N (%)	Girls N (%)
Very Poor	369 (12.4)	206 (13.9)	163 (10.9)
Poor	612 (20.6)	282 (19.1)	330 (22.1)
Medium	972 (32.7)	455 (30.8)	517 (34.6)
Good	724 (24.4)	380 (25.7)	344 (23.0)
Excellent	293 (9.9)	154 (10.4)	139 (9.3)

Percentages for boys and girls are calculated within each sex; total percentages use the full sample (N = 2, 970). Classification was performed using the whole-second cut-points in **Table 6**.

## 4 Discussion

To the best of our knowledge, no age- and sex-specific normative references or percentile-based performance zones for core muscular endurance using the plank exercise test have previously been established for Pakistani adolescents. The present study addresses this gap by developing age- and sex-specific normative references, percentile curves, and percentile-based performance zones for adolescents aged 12 to 16 years in South Punjab, Pakistan, using the LMS method. The internal consistency of the derived references was examined through a back substitution procedure based on a randomly drawn subsample from the same cohort. The findings show that boys recorded higher mean plank times than girls, with no difference detectable at the oldest ages, although the effect sizes were negligible to small (r = 0.02 to 0.14; r = 0.06 overall), with the male advantage concentrated at the upper end of the distribution rather than expressed uniformly across the sample and provide the first population-specific benchmarks for this region.

### 4.1 Sex differences in core muscular endurance

Boys showed significantly higher mean plank times than girls; however, effect sizes were generally small, with plank performance demonstrating a negligible practical effect (rank-biserial r = 0.06). Although the difference was statistically detectable in this large sample, its practical magnitude was limited, and this pattern is broadly consistent with the literature on sex differences in muscular endurance during adolescence. These differences are typically attributed to sex-related differences in muscle mass accumulation and body composition that emerge across the 12–16 year age range (Smith et al., 2019; Carlson, 2022), with boys generally developing greater lean mass relative to body weight, a factor associated with higher isometric endurance performance (Ben Mansour et al., 2021; Carlson, 2022). Laurson et al. (2022) similarly reported that higher trunk lean mass-to-fat mass ratios in boys are associated with superior core endurance performance (Laurson et al., 2022), a pattern broadly consistent with the present findings. However, biological maturation was not assessed; therefore, sex differences likely reflect combined effects of age, sex, and maturation rather than maturation-independent physiological differences (Bchini et al., 2026).

Age-specific analysis showed that significant sex differences were confined to ages 13 and 15 (0.004 and 0.009 respectively), with no detectable difference at age 14 (p = 0.577) or age 16 (p = 0.628). Effect sizes were negligible to small throughout, ranging from r = 0.02 to r = 0.14, and the largest was observed at age 13 rather than at the older ages. The sex difference in mean plank performance therefore did not widen systematically across adolescence in this sample. This pattern is consistent with reports that pubertal change contributes to divergence in core endurance between sexes (Martínez-Romero et al., 2025), but the absence of a monotonic age gradient here, together with the small effect sizes, indicates that any such divergence is not detectable in mean performance within this age range. In the absence of maturation data, the relative contributions of hormonal and behavioural factors cannot be disentangled, and girls’ earlier maturation may partly offset the sex difference during early adolescence.

### 4.2 Normative reference standard

Using the LMS method, this study established age- and sex-specific percentile curves for plank exercise performance. The resulting curves show that median performance increased with age in both sexes, rising from 50 to 61 seconds in boys and from 44 to 66 seconds in girls between ages 12 and 16. Gains at the upper end of the distribution were more pronounced in boys, whose 90th percentile rose steeply from 132 to 226 seconds, than in girls, whose 90th percentile followed a curvilinear course, peaking near age 14 before returning to a similar value at age 16. As these findings are based on smoothed reference curves derived from cross-sectional data, they should be interpreted as descriptive patterns of the fitted model rather than as evidence of longitudinal developmental change.

The back substitution procedure supported the internal consistency of the derived normative references. The average MAPE was 6.69% for boys and 6.04% for girls, within the range conventionally interpreted as indicating accurate agreement (Yang et al., 2025; Khurram, 2026), and all age-specific values fell below 10% with the single exception of 16-year-old boys (10.21%). The close agreement between the development and test sample values suggests that the LMS-derived normative references and plank performance zones are internally stable within this population. However, this check assessed the central tendency (P50) using a subsample drawn from the same cohort used to fit the model. Therefore, it reflects internal consistency rather than external generalizability or criterion validity. Validation in other populations and against health-related outcomes is needed in future research.

Comparisons with available international data indicate that South Punjab adolescents recorded lower plank times than their American counterparts, who averaged approximately 91 seconds for boys and 77 seconds for girls (Ervin, 2013). This disparity is consistent with differences in structured PE provision, access to recreational facilities, developmental factors and broader socioeconomic conditions between the two contexts, which merit further exploration. Adolescents in higher-income settings typically benefit from greater opportunities for structured PA, which is likely to contribute to higher population-level fitness (Graham et al., 2011; Bann et al., 2019). Importantly, despite lower overall mean values, girls in the present sample demonstrated a more consistent pattern of improvement across the age range than has been reported in some international comparisons, which may reflect specific regional or contextual factors that merit further exploration (Boyer et al., 2013).

### 4.3 Establishing muscular endurance performance zones

This study established age- and sex-specific performance zones for the plank exercise test using a five-category classification system based on percentile cut-points (≤P10, P10–P35, P35–P65, P65–P90, and ≥P90), following the methodology adopted from the Chinese National Physical Fitness Evaluation Standard (Jiang, 2004; Yang et al., 2025). To the best of our knowledge, the only previously available criterion-referenced standard for plank performance in youth is that provided by the Canadian Assessment of Physical Literacy (CAPL), which is based on a cohort of 8–12-year-old Canadian children and uses a four-zone classification (Tremblay et al., 2018). The present study extends this framework to a Pakistani adolescent population aged 12–16 years and refines the classification into five zones, providing greater discriminatory precision particularly at the lower end of the distribution where the CAPL’s single “Progressing” zone spans a wide range of ability (Tremblay et al., 2018). The LMS-derived percentile curves also demonstrated satisfactory model fit, supporting the use of these normative references and performance zones for evaluating core muscular endurance in this population. These age- and sex-specific classifications provide a practical tool for physical education teachers, coaches, and health professionals to identify adolescents requiring targeted intervention, monitor changes over time, and support school-based fitness evaluation programmes.

Specifically, what the CAPL describes as a single “Progressing” zone is delineated here into separate “Poor” and “Medium” zones (P10–P35 and P35–P65, respectively), with the Medium zone broadly corresponding to the upper portion of the CAPL’s 17th–65th percentile band. This five-zone approach provides clearer separation between low, moderate, and high-performance groups than the CAPL four-zone model, making the classification more useful for school-based screening and targeted fitness interventions. This finer differentiation enables more nuanced identification of adolescents at intermediate performance levels and supports the design of more precisely targeted interventions.

In the present sample, the majority of participants fell into the “Medium” and “Good” zones, which together accounted for 57.1% of participants, while 33.0% were classified collectively as “Very Poor” or “Poor” and only 9.9% reached the “Excellent” zone. Compared with CAPL data, in which approximately 35% of Canadian children met the “Achieving” or “Excelling” criteria, 34.2% of Pakistani adolescents in this study reached the equivalent “Good” or “Excellent” threshold (Tremblay et al., 2018). This disparity, alongside the cultural and developmental differences between Canadian and Pakistani youth populations (Hadier et al., 2024a), underscores the relevance of population-specific normative references and highlights the need for targeted physical education interventions to improve core muscular endurance among Pakistani youth.

### 4.4 Comparison with FitnessGram and practical considerations

Another study by Laurson et al. (2017) found that approximately 40% of American adolescents met the Healthy Fitness Zone references for the plank exercise as part of the FitnessGram assessment widely used in American schools (Laurson et al., 2017). This rate is higher than observed in the present study, possibly reflecting more extensive participation in structured physical activities and sports programmes in U.S. schools. This disparity underscores the influence of socioeconomic factors and public health policies on physical fitness levels and highlights the necessity of implementing targeted interventions and establishing localized performance criteria to accurately assess and enhance the physical fitness of adolescents in different regions.

The plank test offers clear advantages over traditional core endurance measures such as sit-ups or curl-ups in school settings (Zhang et al., 2024). It requires minimal equipment, is easy to administer, and can be conducted with simple tools such as a stopwatch and mat. Its suitability for population-level assessment under standardised protocols is well documented (Boyer et al., 2013).These strengths support its use in large scale evaluations and in guiding targeted interventions to address fitness disparities.

### 4.5 Age and sex-specific indexes for plank exercise test

The age-related patterns in plank performance observed in this study differed between the sexes and merit careful interpretation. For boys, performance increased with age across the full distribution, but the gains were markedly greater at the upper end than at the centre. The smoothed median (P50) rose from 50 seconds at age 12 to 61 seconds at age 16, while the 90th percentile increased far more steeply over the same interval, from 132 to 226 seconds. This growing separation between the median and the upper percentile indicates a widening of the performance distribution with age, whereby the highest-performing boys improve considerably more than the typical adolescent. Such divergence is consistent with reports that strength and endurance continue to develop through adolescence in boys (Gorzi et al., 2026), and may reflect increasing differentiation in physical activity engagement, training exposure, and body composition as boys progress through this period. Indeed, access to and participation in muscular fitness activities varies considerably among adolescent boys, contributing to uneven developmental trajectories (Reynolds et al., 2022), and some components of fitness are known to depend on lifestyle and environmental factors rather than age alone, further supporting divergence within populations (Iqbal et al., 2024).

For girls, a steady upward trajectory was observed across the central portion of the distribution. The median rose from 44 seconds at age 12 to 66 seconds at age 16, an increase of approximately 22 seconds, with the most pronounced gains occurring in the second half of the age range. In contrast to the boys, the upper percentiles in girls did not diverge from the median; the 90th percentile followed a curvilinear pattern, rising from 126 seconds at age 12 to a peak of 145 seconds at age 14 before declining to 125 seconds at age 16. The distribution therefore became more symmetrical at the oldest ages, with high-performing girls improving at a rate broadly similar to the population median rather than pulling away from it.

This rise in the girls’ central percentiles is consistent with evidence that girls in this age range show meaningful improvement in trunk-related isometric endurance (Andersen, 1994), and that age-related fitness gains in girls tend to occur gradually across adolescence, particularly prior to post-pubertal stabilization (Iqbal et al., 2024). Accordingly, the turnover in the girls’ 90th percentile should not be read as evidence of a genuine decline in high-end performance in late adolescence; because the reference curves are supported by only five whole-year age points and are least constrained at the boundaries of the age range, it is most appropriately interpreted as a boundary effect, and the age-16 upper cut-point should be applied conservatively pending confirmation in data with finer age resolution.

Across the age range, boys generally recorded higher plank performance than girls, and this advantage was most evident at the upper end of the distribution, where the boys’ 90th percentile rose far more steeply with age than the girls’. Notably, this widening male advantage at the upper percentiles was not fully reflected in the group means, which differed only modestly between sexes (rank-biserial r = 0.06). This contrast illustrates why percentile-based references are preferable to mean values when evaluating individual or group fitness: a small number of very high-performing boys can widen the upper tail of the distribution in a way that mean comparisons obscure, and age- and sex-specific percentiles capture this distributional detail directly (Hunter and Senefeld, 2024). Boys are also reported to show larger inter-individual variability in strength and endurance during adolescence, driven by differences in maturation, hormonal status, and activity patterns (Andersen, 1994; Gorzi et al., 2026), which is consistent with the broader upper tail observed here.

The present findings align with international reference data. A Portuguese study reported that median plank times increased with age in both sexes between 10 and 18 years, by 116.9% in boys and 41.4% in girls (Laurson et al., 2022). The same direction was observed here across the narrower 12–16 year window, with median performance rising in both boys and girls. Consistent with the Portuguese pattern of a proportionally larger increase in boys, the present data showed a particularly steep rise in the boys’ upper percentiles, indicating pronounced development among higher-performing boys across this period. These convergent findings reinforce the value of age- and sex-specific normative references for accurate fitness evaluation and underscore the need for longitudinal studies to characterize individual trajectories of core endurance development across adolescence (Laurson et al., 2022).

### 4.6 Study strengths and limitations

#### 4.6.1 Study strengths

This study offers several notable strengths. First, it provides the first age- and sex-specific normative references and percentile-based performance zones for core muscular endurance using the plank exercise test among Pakistani adolescents, addressing a significant gap in regional fitness data. Second, the large, geographically stratified sample of 2, 970 adolescents drawn from three administrative divisions of South Punjab, representing approximately 16.67% of the total eligible schools, enhances the representativeness and generalizability of the findings within this region. Third, the LMS method is a statistically robust approach for constructing percentile curves that accounts for skewness in the data distribution, ensuring that the derived normative references are methodologically sound and consistent with international best practice for normative reference development (Khurram, 2026). Fourth, the use of a back substitution procedure based on a randomly drawn subsample from the same cohort provides an internal consistency check for the LMS-derived normative references; this procedure supports the stability of the central percentile estimates within the study population, while not representing external validation against an independently collected sample. Fifth, the five-zone performance classification system offers greater discriminatory precision than existing four-zone frameworks, enabling more nuanced identification of adolescents at different performance levels, including those at risk of poor muscular endurance outcomes. Finally, the practical feasibility of the plank test in low-resource school settings makes these references directly applicable for use by physical education teachers, coaches, and public health professionals across Pakistan and comparable regional contexts.

#### 4.6.2 Study limitations

Several limitations should be acknowledged. *First*, the study was conducted exclusively in South Punjab, and the normative references derived here may not be directly applicable to adolescents in other regions of Pakistan or in other South Asian populations, given potential differences in socioeconomic conditions, nutritional status, and physical activity levels. Replication across broader geographical settings is recommended before these references are applied at a national level. *Second*, school-level clustering was not modelled in the statistical analyses, and the reported p-values may be slightly anti-conservative as a result. Accordingly, the bootstrap intervals reported here represent lower bounds on the true sampling uncertainty. *Third*, all analyses were based on chronological age, and no measure of biological maturation, such as peak height velocity or somatic maturity estimation, was included. This is important because maturational timing varies considerably between individuals aged 12–16 years and may influence fitness performance independently of chronological age (Bchini et al., 2026). As recent youth fitness assessment literature emphasizes, integrating biological maturity can improve the interpretation of adolescent fitness results and help distinguish chronological age effects from maturational influences (Bchini et al., 2026). This limitation is shared by many large-scale field-based fitness reference systems, which rely on chronological age and sex for practical scalability (Tomkinson et al., 2018; Niessner et al., 2020). Nonetheless, future normative work incorporating maturity offset would allow maturation-adjusted references and a clearer separation of chronological from maturational effects.

*Fourth*, age was recorded in completed years rather than in months or decimal years, so the reference curves were supported by five discrete age points. This limits the resolution with which age-related change can be modelled and the precision of percentile estimates at the boundaries of the age range and in the upper tail, where subgroup sizes are smaller; the reference values should therefore be interpreted at the level of whole-year age groups. *Fifth*, the plank test assesses a single dimension of core muscular endurance, isometric anterior core endurance, and does not capture lateral or posterior core endurance, or dynamic core stability; a comprehensive assessment of trunk muscular fitness would therefore require a battery of tests. *Sixth*, direct test-retest or inter-rater reliability was not assessed within the present study. Although standardized protocols, a training workshop for all assessors, and pre-test familiarization were implemented, the absence of internal reliability data limits the ability to quantify measurement error in this specific sample.

*Seven*, the absolute plank hold time was used as the primary outcome without adjustment for body mass or body composition, as the objective was to establish chronological-age- and sex-referenced norms rather than to model the determinants of performance. Although body mass is known to influence weight-bearing endurance tasks, body-composition-adjusted reference values would require dedicated study and were beyond the scope of the present normative work. Finally, Data collection in 2020 preceded nationwide COVID-19 school closures, providing a clean pre-pandemic reference that no later collection could reconstruct and a benchmark against which post-pandemic change in this population can be assessed. These norms should be revisited against post-pandemic data as it becomes available. The cross-sectional design precludes causal inference or the observation of individual trajectories, and longitudinal studies in this population remain a priority.

### 4.7 Recommendations for future research

Future research in this area should address several of the limitations identified in the present study. Longitudinal investigations are needed to track the development of core muscular endurance across the full adolescent period and to examine how individual trajectories of change relate to broader health outcomes, including lower back pain, postural health, and physical activity levels. Studies incorporating measures of biological maturation, such as peak height velocity assessment or somatic maturity estimation, would allow more precise examination of the relationship between maturational status and core endurance performance and would help disentangle chronological age effects from maturational influences.

Future normative studies should also develop body-composition-adjusted reference values, for example by stratifying percentiles across BMI categories, to clarify the independent contribution of adiposity to core endurance performance. Additionally, future research should examine the influence of nutritional status, physical activity levels, screen time, and socioeconomic factors on core muscular endurance in Pakistani adolescents, as these variables were not captured in the present study but are likely to be important determinants of fitness in this context. Finally, intervention studies evaluating the effectiveness of targeted core endurance training programmes within school physical education settings would provide actionable evidence for improving fitness outcomes among Pakistani youth.

## 5 Conclusion

This study established the first age- and sex-specific normative references and percentile-based performance zones for plank exercise performance among adolescents aged 12–16 years in South Punjab, Pakistan, addressing an important gap in regional fitness data. Median plank performance increased with age in both sexes, while gains at the upper end of the distribution were more pronounced in boys, whose 90th percentile rose steeply across the age range. Boys generally recorded higher plank times than girls, particularly at the upper percentiles. Most participants were classified within the “Medium” or “Good” performance zones. These normative references provide a practical framework for identifying adolescents with lower levels of core muscular endurance who may benefit from targeted physical education interventions.

The back substitution procedure indicated acceptable agreement between the LMS-derived median reference estimates obtained from the complete dataset and those from a randomly selected subsample (average MAPE 6.69% for boys and 6.04% for girls), supporting the internal consistency of the derived normative references within this population. These benchmarks may serve as practical reference tools for PE teachers, coaches, and health professionals in Pakistan and comparable settings. However, because this procedure assessed internal consistency rather than external generalizability, their applicability beyond South Punjab should be confirmed through external validation in independent populations and against relevant health-related outcomes before broader implementation.

## Data Availability

The raw data supporting the conclusions of this article will be made available by the authors, without undue reservation.
