# Supplementary material for: Age- and sex-specific normative references and performance zones for core muscular endurance in adolescents aged 12–16 years: a cross-sectional study
Source: Front Physiol. 2026 Oct 20;17:1802720. doi: 10.3389/fphys.2026.1802720 (PMC13727477; doi:10.3389/fphys.2026.1802720)
Supplement: Supplementary file 1 [file DataSheet1.pdf]

Supplementary Material

The adequacy of the sex-specific LMS/GAMLSS reference models was evaluated using worm plots and Q–Q plots of the normalised (randomised) quantile residuals. In a correctly specified model these residuals follow a standard normal distribution; departures indicate misfit in the modelled location, scale, skewness, or kurtosis.

For both boys and girls, the residuals conformed closely to the standard normal distribution, with means of approximately zero, variances of approximately one, and negligible skewness. In the worm plots, the residual points for every age stratum remained within the 95% confidence bands, with no systematic curvature, trend, or S-shaped pattern that would indicate inadequate modelling of the mean, dispersion, or shape of the distribution across age. In the Q–Q plots, the points fell along the reference line through the central portion of the distribution, with only minor deviations confined to the extreme tails, as expected given the small number of observations at the highest percentiles.

Taken together, these diagnostics indicate that the Box-Cox t distribution, with each LMS parameter modelled as a smooth function of age, provided an adequate fit to the plank performance data for both sexes, supporting the validity of the derived normative reference curves and percentile-based performance zones.

(a) Worm plot — Boys

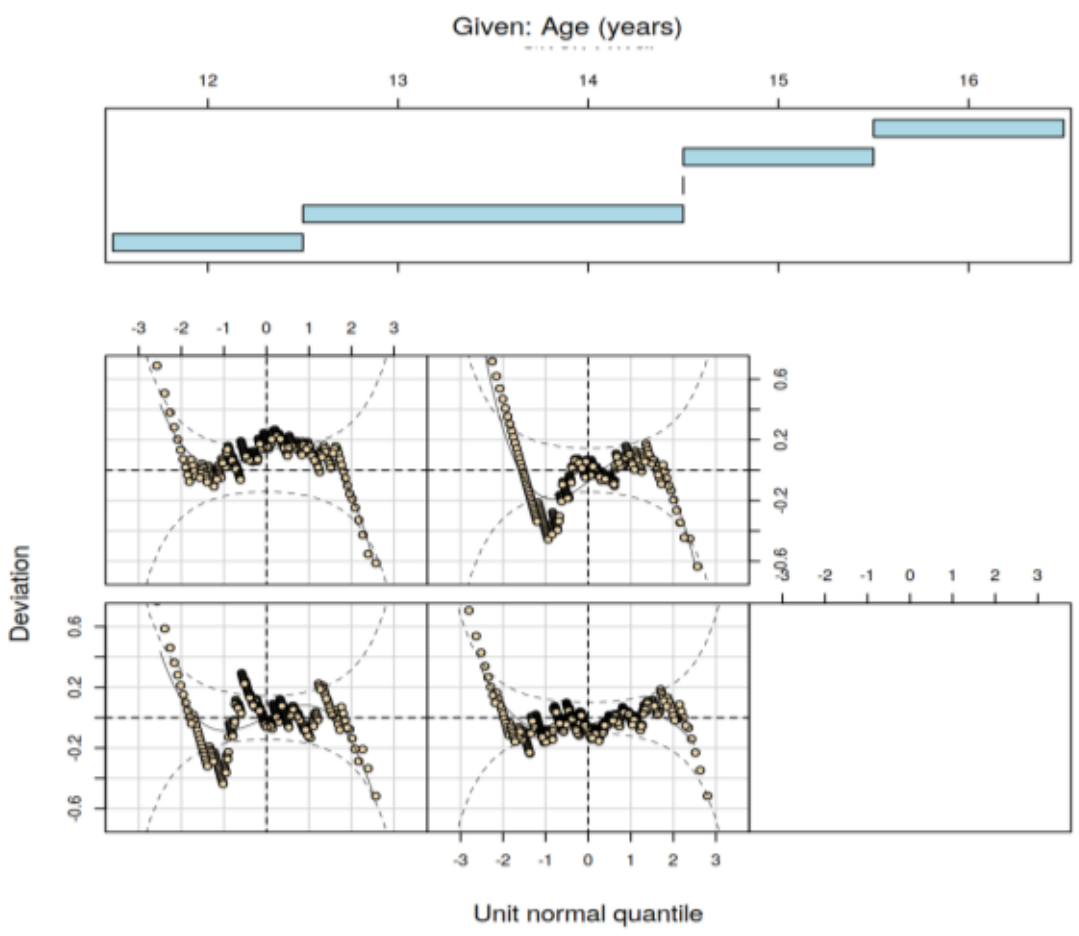

(b) Q-Q plot — Boys

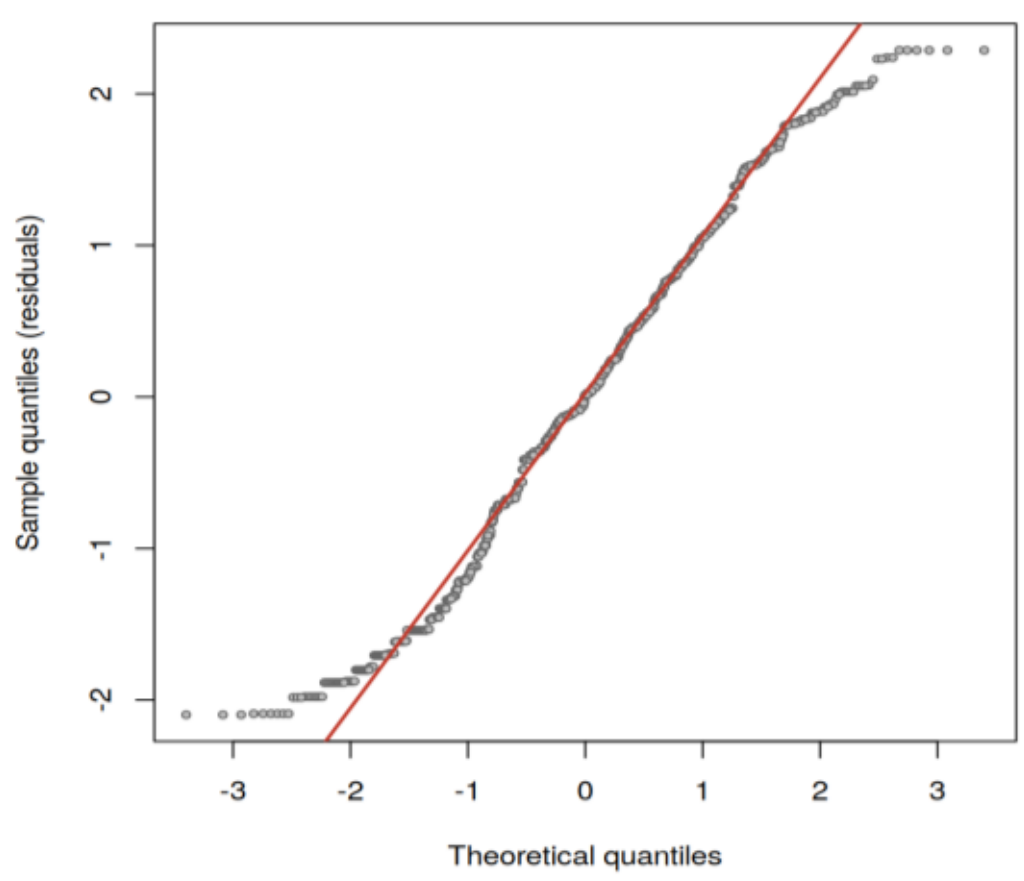

(c) Worm plot — Girls

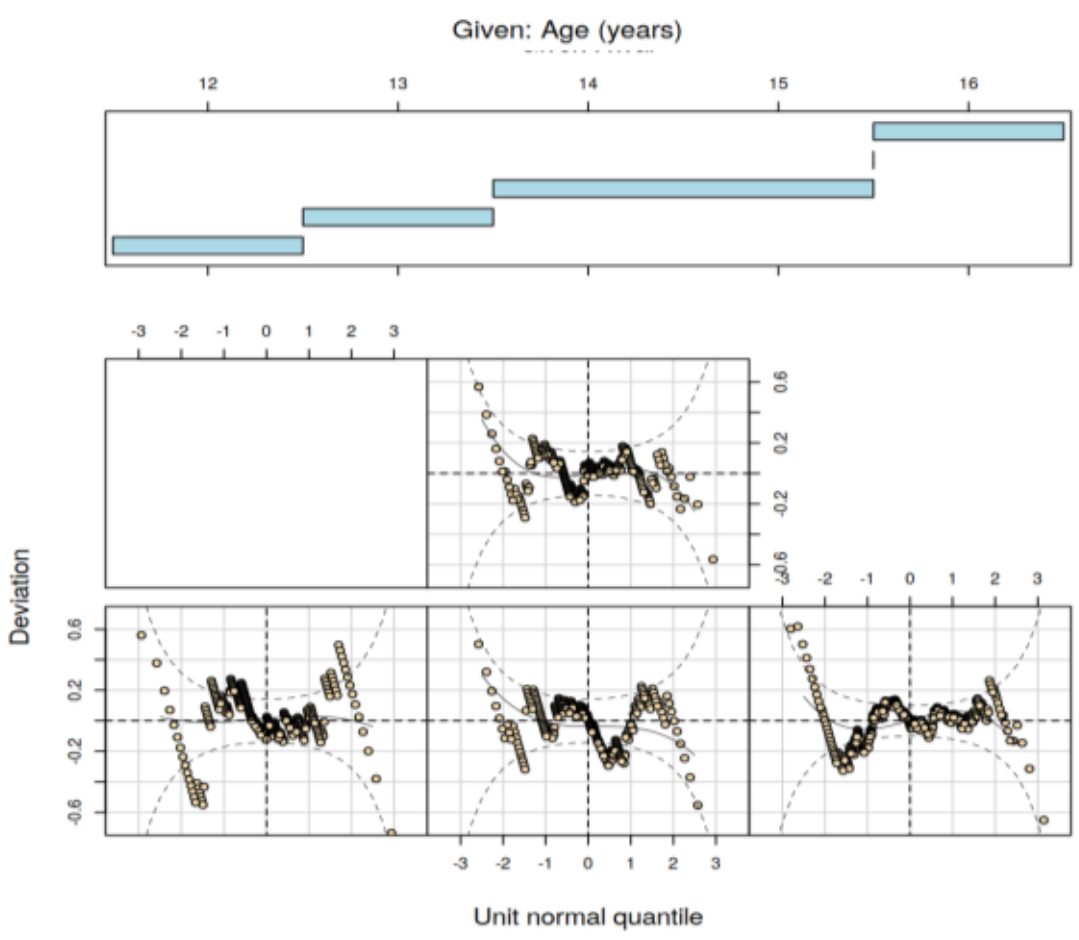

(d) Q-Q plot — Girls

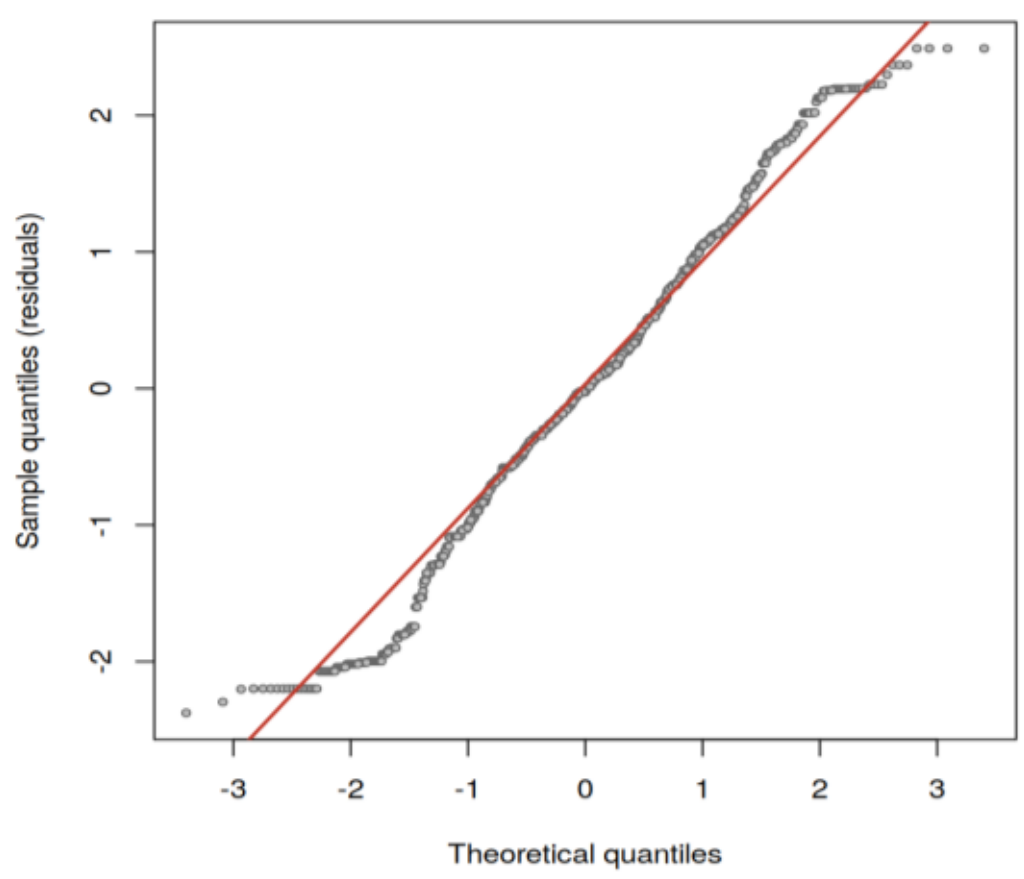

Supplementary Figure S1: Model diagnostics for the age- and sex-specific LMS reference curves, shown separately for boys (top row) and girls (bottom row). Panels (a) and (c) present worm plots of the normalised quantile residuals stratified into age intervals across 12–16 years; points falling within the elliptical 95% confidence bands indicate adequate agreement between the fitted Box-Cox t distribution and the observed data within each age stratum. Panels (b) and (d) present Q–Q plots of the normalised quantile residuals against the standard normal distribution, with the reference line shown in red. For both sexes the residuals conformed closely to normality (boys: mean = 0.00, variance = 1.00, skewness = −0.01; girls: mean = 0.00, variance = 1.00, skewness = −0.01), supporting the adequacy of the fitted reference curves.
