# Supplementary material for: Age- and sex-specific normative references and performance zones for core muscular endurance in adolescents aged 12–16 years: a cross-sectional study
Source: Front Physiol. 2026 Oct 20;17:1802720. doi: 10.3389/fphys.2026.1802720 (PMC13727477; doi:10.3389/fphys.2026.1802720)
Supplement: Supplementary file 2 [file Table1.docx]

**STROBE Statement**

*Checklist of items that should be included in reports of cross-sectional studies*

| **Item** | **Topic** | **STROBE recommendation** | **Where addressed in the manuscript** | **Page** |
| --- | --- | --- | --- | --- |
| **Title and abstract** |  |  |  |  |
| 1a | Title and abstract | Indicate the study design with a commonly used term in the title or the abstract | Title and Abstract identify the study as cross-sectional | 1 |
| 1b |  | Provide in the abstract an informative and balanced summary of what was done and what was found | Abstract: structured summary of design, sample, measures, LMS-derived norms and zone distribution | 2 |
| **Introduction** |  |  |  |  |
| 2 | Background/rationale | Explain the scientific background and rationale for the investigation being reported | Introduction, paragraphs 1-4 | 3 |
| 3 | Objectives | State specific objectives, including any prespecified hypotheses | Introduction, final paragraph (study aims) | 5 |
| **Methods** |  |  |  |  |
| 4 | Study design | Present key elements of study design early in the paper | Methods, opening paragraph (cross-sectional design, data collected 2020) | 5 |
| 5 | Setting | Describe the setting, locations, and relevant dates, including periods of recruitment, exposure, follow-up, and data collection | Methods, opening paragraph: high schools in the Multan, Bahawalpur and D.G. Khan divisions of South Punjab; data collected in 2020 | 5 |
| 6a | Participants | Give the eligibility criteria, and the sources and methods of selection of participants | Methods - Sampling; Methods - Participation Criteria; Figure 1 | 7 |
| 6b |  | (Matched studies) Give matching criteria and number of exposed and unexposed | Not applicable - cross-sectional design without matching | N/A |
| 7 | Variables | Clearly define all outcomes, exposures, predictors, potential confounders, and effect modifiers. Give diagnostic criteria, if applicable | Methods - Measures and Procedure (plank hold time, height, weight, BMI); age and sex as stratifying variables | 8 |
| 8 | Data sources/ measurement | For each variable of interest, give sources of data and details of methods of assessment (measurement). Describe comparability of assessment methods if there is more than one group | Methods - Measures and Procedure; Methods - Study Procedure (standardised protocol, trained assessors, hold time recorded in whole seconds) | 8-9 |
| 9 | Bias | Describe any efforts to address potential sources of bias | Methods - Study Procedure (standardised testing conditions and familiarisation); Methods - Participation Criteria (exclusion criteria); Discussion - Limitations | 7 |
| 10 | Study size | Explain how the study size was arrived at | Methods - Sampling (sample size formula, design effect, and rationale for exceeding the calculated minimum) | 6 |
| 11 | Quantitative variables | Explain how quantitative variables were handled in the analyses. If applicable, describe which groupings were chosen and why | Methods - Statistical Analysis; age analysed in completed years across five strata; plank time modelled continuously and classified into five performance zones (Table 6) | 10-13 |
| 12a | Statistical methods | Describe all statistical methods, including those used to control for confounding | Methods - Statistical Analysis (Kolmogorov-Smirnov test, Mann-Whitney U test, rank-biserial correlation); Methods - LMS Method and Its Application (BCCG distribution within the GAMLSS framework) | 13 |
| 12b |  | Describe any methods used to examine subgroups and interactions | Methods - Statistical Analysis; all analyses stratified by age and sex | 10-13 |
| 12c |  | Explain how missing data were addressed | Methods - Sampling (non-responsive and incomplete responses excluded prior to analysis; the analytic sample of n = 2,970 contains complete data for all analysed variables) | 13 |
| 12d |  | If applicable, describe analytical methods taking account of sampling strategy | Methods - Statistical Analysis; Discussion - Limitations (school-level clustering not modelled; no school identifier was retained in the analytic file) | 13 |
| 12e |  | Describe any sensitivity analyses | Methods - Back substitution procedure; Methods - Statistical Analysis (non-parametric bootstrap, 1,000 replicates per sex); Supplementary Tables S2 and S3 | 10-13 |
| **Results** |  |  |  |  |
| 13a | Participants | Report numbers of individuals at each stage of study | Figure 1 (sampling flow chart); Results, opening paragraph; Table 1 | 7 |
| 13b |  | Give reasons for non-participation at each stage | Figure 1; Methods - Participation Criteria | 7 |
| 13c |  | Consider use of a flow diagram | Figure 1 | 7 |
| 14a | Descriptive data | Give characteristics of study participants and information on exposures and potential confounders | Tables 1 and 2 (age, sex, height, weight and BMI) | 13 |
| 14b |  | Indicate number of participants with missing data for each variable of interest | Methods - Sampling; the analytic sample contains complete data for all analysed variables | 6 |
| 15 | Outcome data | Report numbers of outcome events or summary measures | Table 3 (age- and sex-specific plank performance); Table 5 (LMS parameters and percentile values) | 14 |
| 16a | Main results | Give unadjusted estimates and, if applicable, confounder-adjusted estimates and their precision. Make clear which confounders were adjusted for and why they were included | Tables 5 and 6; Supplementary Table S2 (bootstrap 95% confidence intervals for all tabulated percentiles) | 17-18 |
| 16b |  | Report category boundaries when continuous variables were categorized | Table 6 (performance zone cut-points, reported in whole seconds) | 18 |
| 16c |  | If relevant, consider translating estimates of relative risk into absolute risk for a meaningful time period | Not applicable - normative reference study; no risk estimates reported | N/A |
| 17 | Other analyses | Report other analyses done - eg analyses of subgroups and interactions, and sensitivity analyses | Table 4 and Results (back-substitution procedure); Supplementary Table S2 (bootstrap confidence intervals); Supplementary Table S3 (model-based versus empirical percentiles) | 15 |
| **Discussion** |  |  |  |  |
| 18 | Key results | Summarise key results with reference to study objectives | Discussion, opening paragraph | 19 |
| 19 | Limitations | Discuss limitations of the study, taking into account sources of potential bias or imprecision. Discuss both direction and magnitude of any potential bias | Discussion - Limitations (cross-sectional design, school-level clustering, absence of maturation data, reduced precision of the age-16 estimates) | 19-25 |
| 20 | Interpretation | Give a cautious overall interpretation of results considering objectives, limitations, multiplicity of analyses, results from similar studies, and other relevant evidence | Discussion - Sex Differences in Core Muscular Endurance; Discussion - Normative Reference Standard; comparison with international reference data | 19-22 |
| 21 | Generalisability | Discuss the generalisability (external validity) of the study results | Discussion - Limitations; Conclusion (applicability to South Punjab adolescents and caution regarding extrapolation) | 25-28 |
| **Other information** |  |  |  |  |
| 22 | Funding | Give the source of funding and the role of the funders for the present study and, if applicable, for the original study on which the present article is based | Financial Disclosure Statement: this research received no external funding | 29 |
