# Supplementary material for: Age- and sex-specific normative references and performance zones for core muscular endurance in adolescents aged 12–16 years: a cross-sectional study
Source: Front Physiol. 2026 Oct 20;17:1802720. doi: 10.3389/fphys.2026.1802720 (PMC13727477; doi:10.3389/fphys.2026.1802720)
Supplement: Supplementary file 3 [file Table2.docx]

**Supplementary Table S2.** Bootstrap 95% confidence intervals for LMS-derived percentiles of plank performance (seconds), by age and sex

| **Age (years)** | **P3** | **P10** | **P35** | **P50** | **P65** | **P90** |
| --- | --- | --- | --- | --- | --- | --- |
| **Boys (n = 1,477)** |  |  |  |  |  |  |
| 12 | 10  (9–12) | 17  (16–20) | 37  (34–41) | 50  (46–56) | 68  (62–75) | 132  (121–145) |
| 13 | 12  (11–13) | 20  (18–21) | 40  (38–43) | 55  (51–58) | 74  (69–79) | 147  (137–157) |
| 14 | 12  (11–13) | 20  (18–22) | 42  (39–46) | 58  (53–63) | 80  (74–86) | 166  (153–180) |
| 15 | 10  (9–11) | 18  (17–19) | 42  (39–46) | 60  (56–65) | 86  (79–92) | 191  (178–205) |
| 16 | 6  (6–7) | 14  (12–16) | 40  (34–47) | 61  (52–71) | 92  (80–105) | 226  (200–252) |
| **Girls (n = 1,493)** |  |  |  |  |  |  |
| 12 | 12  (11–14) | 18  (16–20) | 33  (30–36) | 44  (40–48) | 59  (54–65) | 126  (110–142) |
| 13 | 11  (11–13) | 17  (16–19) | 34  (32–36) | 46  (44–49) | 64  (61–68) | 144  (134–157) |
| 14 | 11  (10–12) | 18  (17–20) | 37  (35–40) | 51  (48–55) | 70  (65–75) | 145  (134–158) |
| 15 | 11  (10–13) | 20  (18–22) | 43  (40–46) | 58  (54–61) | 76  (71–80) | 135  (126–142) |
| 16 | 12 *  (4–15) | 25 *  (18–28) | 52  (47–56) | 66  (61–71) | 82  (77–88) | 125  (118–133) |

**Note:** Values are the fitted percentile in whole seconds with the bootstrap 95% confidence interval in parentheses. Intervals were obtained by drawing 1,000 resamples of the full sample with replacement within each sex, refitting the BCCG model to each resample, and recomputing percentiles at every age; percentile-based limits were taken from the resulting distributions. All 1,000 replicates converged for both sexes. Most estimates have relative interval widths of 11–20% of the fitted value. Precision is lowest at age 16 in both sexes, reflecting the reduced constraint on the quadratic age term at the boundary of the age range. Asterisks (*) denote estimates with a relative interval width of 40% or more; the girls’ P3 value at age 16 (relative width 92%) should be regarded as indicative only and is not recommended for individual classification.
