# Supplementary material for: Age- and sex-specific normative references and performance zones for core muscular endurance in adolescents aged 12–16 years: a cross-sectional study
Source: Front Physiol. 2026 Oct 20;17:1802720. doi: 10.3389/fphys.2026.1802720 (PMC13727477; doi:10.3389/fphys.2026.1802720)
Supplement: Supplementary file 4 [file Table3.docx]

**Supplementary Table S3.** Model-based and empirical percentiles of plank performance (seconds), by age and sex

**Panel A. Fitted versus empirical percentiles**

| **Age (years)** | **P3** | **P10** | **P35** | **P50** | **P65** | **P90** |
| --- | --- | --- | --- | --- | --- | --- |
| **Boys** |  |  |  |  |  |  |
| 12 | 10 / 11  (-1) | 17 / 14  (+3) | 37 / 42  (-5) | 50 / 48  (+2) | 68 / 72  (-4) | 132 / 151  (-19) |
| 13 | 12 / 12  (+0) | 20 / 21  (-1) | 40 / 41  (-1) | 55 / 52  (+3) | 74 / 74  (+0) | 147 / 162  (-15) |
| 14 | 12 / 11  (+1) | 20 / 15  (+5) | 42 / 42  (+0) | 58 / 52  (+6) | 80 / 71  (+9) | 166 / 148  (+18) |
| 15 | 10 / 10  (+0) | 18 / 17  (+1) | 42 / 46  (-4) | 60 / 71  (-11) | 86 / 100  (-14) | 191 / 211  (-20) |
| 16 | 6 / 10  (-4) | 14 / 10  (+4) | 40 / 41  (-1) | 61 / 63  (-2) | 92 / 88  (+4) | 226 / 250  (-24) |
| **Girls** |  |  |  |  |  |  |
| 12 | 12 / 10  (+2) | 18 / 21  (-3) | 33 / 34  (-1) | 44 / 40  (+4) | 59 / 53  (+6) | 126 / 110  (+16) |
| 13 | 11 / 11  (+0) | 17 / 20  (-3) | 34 / 37  (-3) | 46 / 47  (-1) | 64 / 53  (+11) | 144 / 174  (-30) |
| 14 | 11 / 10  (+1) | 18 / 18  (+0) | 37 / 43  (-6) | 51 / 50  (+1) | 70 / 66  (+4) | 145 / 144  (+1) |
| 15 | 11 / 10  (+1) | 20 / 16  (+4) | 43 / 46  (-3) | 58 / 56  (+2) | 76 / 74  (+2) | 135 / 132  (+3) |
| 16 | 12 / 11  (+1) | 25 / 30  (-5) | 52 / 48  (+4) | 66 / 66  (+0) | 82 / 85  (-3) | 125 / 120  (+5) |

**Note:** Each cell shows the fitted percentile / the empirical percentile, with the difference in parentheses. Empirical percentiles were computed directly from the observed distribution within each age-by-sex stratum. Mean absolute discrepancies were 1.1 s at P3, 2.9 s at P10, 2.8 s at P35, 3.2 s at P50, 5.7 s at P65 and 15.1 s at P90, so agreement is close across the lower and central percentiles and looser at P90, consistent with the wider bootstrap intervals in Supplementary Table S2. The three largest deviations are the girls’ P90 at age 13 (144 vs 174 s), the boys’ P50 at age 15 (60 vs 71 s) and the boys’ P65 at age 15 (86 vs 100 s). Each arises where the observed sequence is non-monotonic across adjacent ages: the observed medians for boys are 48, 52, 52, 71 and 63 s at ages 12 to 16, and a quadratic function of age fitted to five whole-year points cannot follow a single-year departure of this kind. Smoothing such fluctuations is the intended behaviour of the LMS method rather than a shortcoming of the fit, since it is what allows the reference curves to remain stable across age and transferable to other samples; empirical percentiles computed separately within each stratum would reproduce these fluctuations exactly but would carry the sampling noise of that stratum with them, which is why smoothed rather than empirical values are used as the basis for the reference curves and performance zones. A further consideration is that hold time was recorded in whole seconds and values cluster heavily at the lower boundary of the scale: among boys aged 16, 34 of 291 participants (11.7%) recorded exactly 10 s, so a cut-point placed at or above that value necessarily captures more than the nominal 10% of the stratum irrespective of how the percentile is estimated.

**Panel B. Fitted median, observed median, observed mean and skewness in girls**

| **Age (years)** | **Fitted P50** | **Observed median** | **Observed mean** | **Skewness** |
| --- | --- | --- | --- | --- |
| 12 | 44 | 40 | 64.88 | 2.47 |
| 13 | 46 | 47 | 67.00 | 2.15 |
| 14 | 51 | 50 | 71.35 | 2.15 |
| 15 | 58 | 56 | 70.69 | 1.42 |
| 16 | 66 | 66 | 71.48 | 0.64 |

**Note:** The fitted median tracks the observed median closely at every age. The observed mean rises far less steeply because the distribution becomes progressively less skewed with age: skewness falls from 2.47 at age 12 to 0.64 at age 16, and the ratio of mean to median falls from 1.62 to 1.08. At the younger ages the mean is inflated by a long right tail, whereas by age 16 the distribution is close to symmetric. The divergence between the fitted median and the observed mean therefore reflects the change in distributional shape rather than any discrepancy in model fit.
